# Effect of Adult Male Diet on Fertilization and Hatching in an Insect

**DOI:** 10.17912/micropub.biology.001074

**Published:** 2024-01-31

**Authors:** Evan Force, Caroline Suray, Matthieu Dacher, Stéphane Debernard

**Affiliations:** 1 Sorbonne Université, Université Paris-Est Créteil, INRAE, CNRS, IRD, Institute for Ecology and Environmental Sciences of Paris, iEES Paris, F-78026, Versailles, France; 2 Sorbonne Université, Université Paris-Est Créteil, INRAE, CNRS, IRD, Institute for Ecology and Environmental Sciences of Paris, iEES Paris, F-75005, Paris, France

## Abstract

As in other animals, diet is known to influence insect reproduction, and its impact has been intensively investigated in females. In our study, we examined the effects of various diets on male reproductive success in the moth *Agrotis ipsilon,* a pest of many crops. Our experiments showed an increase in the rates of fertilization and hatching when males fed with various sugars (sucrose, fructose, and glucose) supplemented with sodium. Such results provide valuable initial information on the nutritional ecology of male moths and could serve to the development of nutritional attractants for the management of crop pests.

**Figure 1. Fertilization and hatching of fertilized eggs according to adult male diet:**
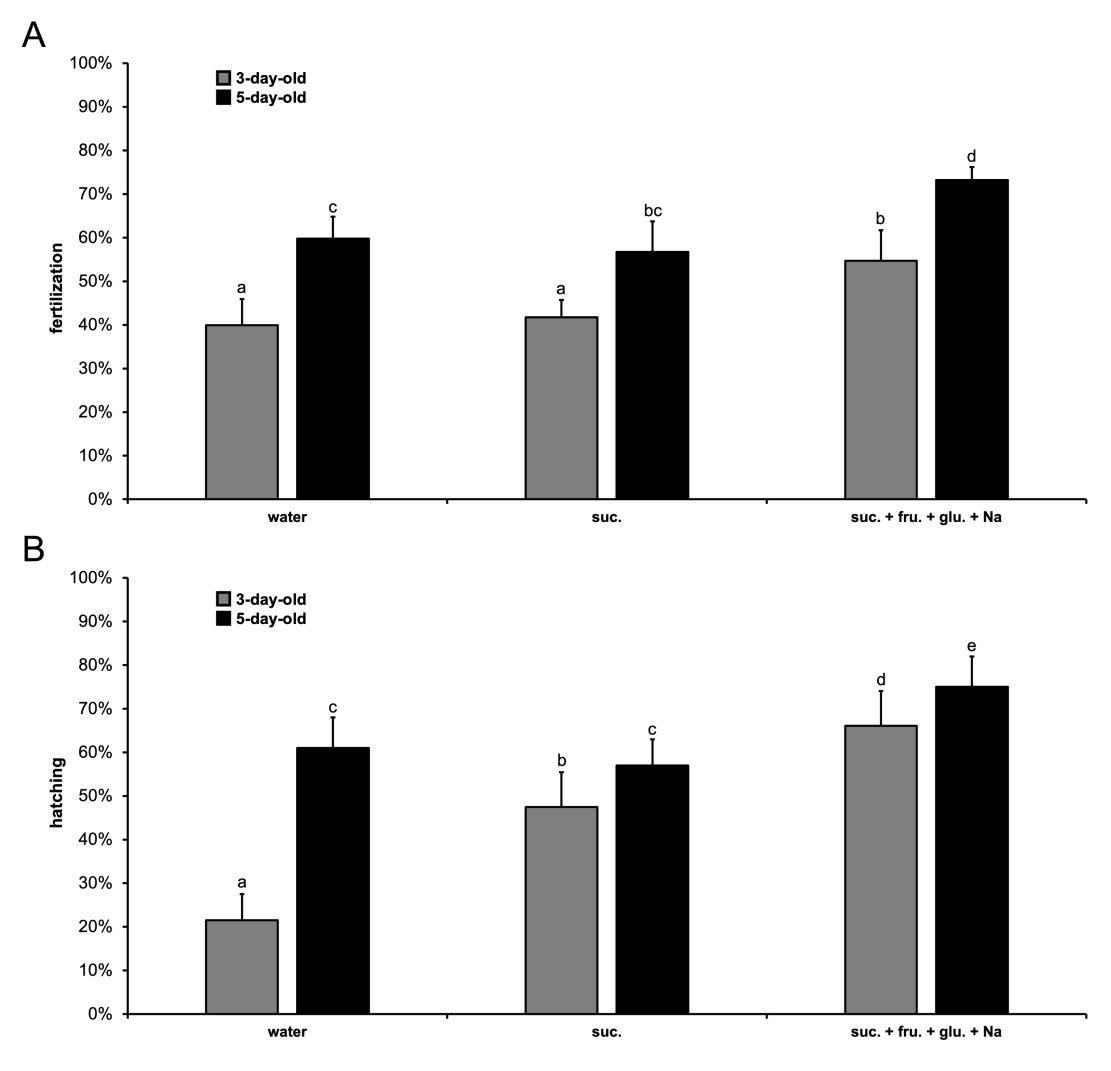
The fertilization and hatching rates were assessed on males fed during three (3-day-old, grey) or five (5-day-old, black) days after emergence. Values correspond to the mean rate ± SEM (n = 15 for all the diets except for the suc. + fru. + glu. + Na diet where n _D3_ = 13 and n _D5_ = 14). Letters represent the statistical differences after a Chi-squared test ( *p < 0.05* ). ( **A** ) Results for fertilization rate depending on adult male diet. ( **B** ) Results for hatching rate according to adult male diet.

## Description

For all living organisms, nutrients are crucial by providing the energy for the achievement of vital physiological functions, especially reproduction (Brooker et al., 2013; Duxbury & Chapman, 2020; Eldrigde & Krapu, 1988; García-González et al., 2016; Geister et al., 2008; Katsuki et al., 2012; Naya et al., 2007). In insects, the impact of food on reproductive fitness has been extensively explored in females. For instance, in female Lepidoptera, a diet rich in sugars such as flower nectar was found to increase oviposition, fecundity, and offspring longevity (Geister et al., 2008; He et al., 2022, 2021; Marchioro & Foerster, 2013; O’Brien et al., 2004; Su et al., 2022). Conversely, less attention has been paid to relationships between feeding and reproduction in males both in Lepidoptera and in other insect orders. In addition to the nectar diet, males of many Lepidoptera species display a puddling behavior to collect sodium (Molleman, 2010; Smedley & Eisner, 1996) . Sodium would have a role in reproduction (Mitra et al., 2016) , and also in nutrition by facilitating the absorption of sugars in the gut (McLean & Caveney, 1993) . This would allow an additional supply of energy essential for the growth and reproduction of Lepidoptera. Interestingly, a recent study demonstrated in the male moth *Agrotis ipsilon* that a diet consisting of sucrose, fructose, and glucose along with sodium induced an acceleration in the development of the reproductive system during sexual maturation (Force et al., 2023) .

In the male *A. ipsilon* , sexual maturation occurs through adult life, and more precisely between the third and fifth days post-emergence under standard rearing conditions with 12% sucrose (Gemeno & Haynes, 2000) . During this period, the reproductive system is subject to morpho-anatomical and functional modifications (Force et al., 2023) . The testes decrease in volume and the sperm bundles migrate from these organs towards the duplex to be stored in the spermatophore, which is then transferred to the female during copulation. Concomitantly, the accessory sex glands grow in length and increase their biosynthetic activity of seminal substances that are required for the formation of the spermatophore, the survival of the spermatozoa and their transit through the female genital tract (Duportets et al., 1998; Force et al., 2023) .

To gain more information about the influence of the feeding on the reproductive success of the adult male *A. ipsilon* , we have evaluated the rates of fertilization and hatching when virgin and sexually mature 3-day-old females (all fed with sucrose) were mated with virgin males fed during 3 or 5 days after emergence with one of the following three diets: water, sucrose, and sucrose + fructose + glucose + sodium. We excluded fructose or glucose diets because it has been demonstrated in males *A. ipsilon* that the gustatory responsiveness to glucose and fructose, measured by the proboscis extension response, is at a very low level compared to sucrose alone and the mixture of sucrose + fructose + glucose. These results suggest that males would not consume glucose and fructose alone (Hostachy et al., 2019) . Interestingly, Hostachy et al. (2019) also showed that the supplementation of glucose to various sugar mixtures did not modify the male gustatory response. Additionally, the nectar sucrose-to-glucose/fructose ratio was found to be higher in plants that bloom at night (Tiedge & Lohaus, 2017) . Knowing that *A. ipsilon* is a nocturnal insect, it is probable that males fed nectars in which sucrose is the dominant sugar and that its presence is indispensable for flower nectar consumption.

We first examined the fertilization rate as a function of the adult male diet ( **Fig. 1A** ). For 3-day-old (D3) males, the rate fertilization is around 40% with water or sucrose (Chi-squared test, χ² = 0.93, *p = 0.334* ) and is higher, approximatively of 55%, with a diet composed of sucrose, fructose, glucose and sodium (Chi-squared test, χ² ≥ 46.36, *p < 0.0001* ). Similar effects were observed for 5-day-old (D5) males with a fertilization level of 73% for a diet comprising the three sugars and sodium that is more elevated (Chi-squared test, χ² ≥ 57.70, *p < 0.0001* ) than with water or sucrose (around 59%) (Chi-squared test, χ² = 2.78, *p = 0.096* ). In addition, whatever the tested diet, the fertilization rate increases by approximately 20% between D3 and D5 males (Chi-squared test, χ² ≥ 66.92, *p < 0.0001* ). Interestingly, the fertilization rate with D3 males fed three sugars supplemented with sodium is identical to that of D5 males fed with sucrose (Chi-squared test, χ² = 1.09, *p = 0.295* ).

We then assessed the hatching rate of fertilized eggs according to the adult male diet ( **Fig. 1B** ). For D3 males, the hatching rate is only 22% with water and is much lower than with two sugary diets (Chi-squared test, χ² ≥ 157.74, *p < 0.0001* ). The hatching rate is the highest with three sugars and sodium with an approximative value of 65% and is superior to 15% compared to sucrose diet (Chi-squared test, χ² = 52.68, *p < 0.0001* ). For D5 males, the hatching rate is around 60% for water and sucrose (Chi-squared test, χ² = 2.19, *p = 0.140* ), while it is more elevated (equal to 75%) with three sugars and sodium (Chi-squared test, χ² ≥ 36.79, *p < 0.0001* ). In addition, independently of the tested diet, the hatching rate of fertilized eggs increases between D3 and D5 males (Chi-squared test, χ² ≥ 4.83, *p ≤ 0.028* ). Interestingly, the number of fertilized eggs for D3 males fed with three sugars supplemented with sodium is higher than for D5 males fed with water or sucrose (Chi-squared test, χ² ≥ 11.05, *p < 0.001* ).

Finally, our results highlight that the fertilization and the hatching of fertilized eggs are more efficient for males fed with various sugars (sucrose, fructose, glucose) supplemented with sodium during three- or five-days post-emergence in comparison to males fed with water or sucrose. In addition, it is interesting to note that 3-day-old males fed with various sugars and sodium have a similar fertilization capacity to 5-day-old males fed with water or sucrose while generating more larvae. Therefore, a diet composed of a variety of sugars and sodium has a significant positive impact on reproduction by promoting the reproductive fitness of 3-day-old males in *A. ipsilon* . It seems reasonable to believe that the influence of such a diet might be accompanied by modulatory effects on the reproductive system especially on the testes and the accessory sex glands.

In Lepidoptera males, the testicular differentiation and the production of spermatozoa mainly take place before the emergence of adults (Seth et al., 2023) . Therefore, it is unlikely that the increase in fertilization is due to a higher sperm production according to the diet. A recent work by Force et al. (2023) demonstrated in virgin *A. ipsilon* males that a diet composed of various sugars (sucrose, fructose, glucose) with sodium accelerated the migration of sperm bundles from the testes to the duplex within the first three days after emergence. Such a phenomenon might in part be responsible for the higher fertilization rate for 3-day-old males fed with various sugars along with sodium in comparison to males fed water or sucrose. It has also been previously reported that regardless of the diet, the number of sperm bundles in the duplex did not increase between the third and the fifth post-emergence day, while in this present study, we nevertheless observed a greater fertilization capacity of 5-day-old males compared to 3-day-old males for all tested diets. All the above-mentioned data led us to hypothesize that the stimulatory effect of a sugar-rich diet with sodium on fertilization would not only result in a higher number of sperm bundles stored in the spermatophore delivered to the female during mating.

In adult male *A. ipsilon* , the accessory sex glands are significantly impacted by diet (Force et al., 2023) . Their growth in length as well as their biosynthetic activity are greatly increased when males are fed with various sugars and sodium for the first three or five days of adult life. Furthermore, it is well-known that accessory sex glands play a major role in insect reproduction (Gillott, 2003; Jin & Gong, 2001; Wu et al., 2023) . These reproductive organs are involved in the formation of the spermatophore in Lepidoptera males (Srinivasan et al., 1998) , and the seminal fluid produced by the accessory sex glands contains a multitude of bioactive proteins which are also transferred to the female during mating (Wu et al., 2023) . The seminal proteins of the accessory sex glands are required for nourishment, activation and capacitation of spermatozoa until fertilization (Adiyodi, 1988; Gillott, 2003; Hartmann & Loher, 1999; Heifetz et al., 2001; Neubaum & Wolfner, 1999; Tram & Wolfner, 1999) , and their influence also extends to females by triggering physiological post-mating changes including accelerated egg maturation, enhanced ovulation and egg laying (Gillott, 2003; Hiss & Fuchs, 1972; Jin & Gong, 2001; Park et al., 1998; Srinivasan et al., 1998) .

Therefore, it is highly conceivable that the positive impact of a sugar-rich diet with sodium on the reproductive success of adult male *A. ipsilon* is mediated by promoting effects on the migration of sperm bundles from testes to duplex as well as on the development and secretory activity of the accessory sex glands. To confirm the existence of such interactions between diet and reproduction, it will be interesting, in future experiments, to perform a comparative analysis of the proteome of seminal fluid as a function of male diet in *A. ipsilon* .

## Methods

**Insects and Dietary Regimens.** Our work was carried out on *Agrotis ipsilon* (Noctuidae, Lepidoptera) after their emergence. This insect is native to the south of France. They were bred in our laboratory at INRAE in Versailles, France. Rearing was carried out according to the protocol described by Force et al. (2023); males and females were separated from pupation so that males are completely naïve regarding female sex-pheromone. Just after the emergence, females were fed with standard rearing condition, either 12% sucrose (mass/mass or 13.6% mass/volume); whereas males were subjected to different diets. The diet effects were examined in males on the third day (D3) and fifth day (D5) of adult life. These days correspond, respectively, to the beginning and the end of sexual maturation in the standard rearing conditions (Gemeno & Haynes, 2000) . The diet water is a negative control (fasting males). The other two conditions contained osmosed water in addition to:

· 12% sucrose (noted suc.);

· 4% sucrose with 4% fructose, 4% glucose, and 1% NaCl (noted suc. + fru. + glu. + Na).

These sugars are the major constituents of the flower nectar, the main food source for adult Lepidoptera, with generally 10-15% of the fresh mass for a single sugar or a mixture of three sugars (Roy et al., 2017) . Sodium is an important mineral element in the reproduction of many moths (Molleman, 2010; Smedley & Eisner, 1996) . All reagents (sugars and NaCl) come from Sigma-Aldrich (Saint-Quentin-Fallavier, France).

**Experiment.** In rearing conditions, after emergence, D5 males mate with D3 females. In this experiment, we assessed the fertilization rate as well as the hatching rate based on the diet of males aged 3 or 5 days after emergence. To do so, we presented virgin D3 females (all fed 12% sucrose) to virgin D3 or D5 males fed different previous diets. The couples were made randomly within small transparent circular boxes (7 cm in diameter x 5 cm in height) thus forcing the mating, each closed by a transparent perforated cover. Moreover, the bottom of the boxes was covered with a disk of Wattman filter paper, and a strip of filter paper was also attached to the internal side walls. All couples were maintained for 3 days in an incubator (22 °C, 60-70% relative humidity, photoperiod 16L:8D). The adults could feed themselves using a drinker (2 x 2 x 1 cm) located in the center of the box and filled with a sugar solution (12% sucrose). After 3 days, the couples were separated and the adults were eliminated. In addition, for each circular box, the Wattman filter paper strip was collected to count the eggs. From one of the two ends of the filter paper strip, we recorded the number of fertilized eggs out of a total of 100 eggs counted under a Leica S9D stereomicroscope. Subsequently, each circular box corresponded to a transparent rectangular box (15 cm x 8 cm x 5 cm), closed and ventilated by 2 lateral fine grids, in which the 100 eggs previously counted were deposited. These eggs were incubated (25 °C, 60-70% relative humidity, photoperiod 16L:8D) for 5 days, and the number of larvae that hatched was counted under a Leica S9D stereomicroscope. The hatching rate was calculated for each pair by dividing the number of larvae by the number of fertilized eggs.

**Statistical Analysis.** Data analysis was carried out using the software R Core Team (2021) and RStudio 2021.09.2. Sample size was 13, 14 or 15 (see legend to **Fig. 1** ). Comparisons of fertilization and hatching rates according to diets were made using a Chi-square test. After a significant Chi-square test, pairwise comparisons were adjusted with Holm’s method. The alpha risk was 0.05.

**Ethical Note.** The French legislation for animal welfare is modeled on the European Union (Directive 2010/63/EU) according to which no invertebrate other than cephalopods enjoy ethical protection. However, all manipulations were carried out with care, minimizing stress situations for the animals.
